# Electronic Vapor Product Use Among High School Students — Youth Risk Behavior Survey, United States, 2021

**DOI:** 10.15585/mmwr.su7201a11

**Published:** 2023-04-28

**Authors:** Briana E. Oliver, Sherry Everett Jones, Emily Devora Hops, Carmen L. Ashley, Richard Miech, Jonetta J. Mpofu

**Affiliations:** ^1^Office on Smoking and Health, National Center for Chronic Disease Prevention and Health Promotion, CDC; ^2^Division of Adolescent and School Health, National Center for HIV, Viral Hepatitis, STD, TB Prevention, CDC; ^3^Institute for Social Research, University of Michigan, Ann Arbor, MI; ^4^U.S. Public Health Service Commissioned Corps, Rockville, MD

## Abstract

Commercial tobacco use is the leading cause of preventable disease and death in the United States. Despite declines in overall tobacco product use among youths, disparities persist. This report uses biennial data from the 2015–2021 cycles of the nationally representative Youth Risk Behavior Survey to assess prevalence and trends in electronic vapor product (EVP) use among high school students, including ever use, current use (past 30 days), and daily use. Data from 2021 also included usual source of EVPs among students who currently used EVPs. Overall, in 2021, 36.2% had ever used EVPs, 18.0% currently used EVPs, and 5.0% used EVPs daily, with variation in prevalence by demographic characteristics. Prevalence of ever use and current use of EVPs was higher among female students than male students. Prevalence of ever use, current use, and daily use of EVPs was lower among Asian students than Black or African American (Black), Hispanic, Native Hawaiian or other Pacific Islander, White, and multiracial students. Prevalence of ever use, current use, and daily use of EVPs was higher among bisexual students than among students who were not bisexual. During 2015–2021, although ever use of EVPs decreased overall (from 44.9% to 36.2%) and current use of EVPs was stable overall, daily EVP use increased overall (from 2.0 to 5.0%) and among female (from 1.1% to 5.6%), male (from 2.8% to 4.5%), Black (from 1.1% to 3.1%), Hispanic (from 2.6% to 3.4%), multiracial (from 2.8% to 5.3%) and White (from 1.9% to 6.5%) students. Among students who currently use EVPs, 54.1% usually got or bought EVPs from a friend, family member, or someone else. Continued surveillance of EVP and other tobacco product use is necessary to document and understand youth tobacco product usage. These findings can be used to inform youth-focused tobacco prevention and control strategies at the local, state, tribal, and national levels.

## Introduction

Tobacco product use is the leading cause of preventable disease and death in the United States (*1*). The term “tobacco product” in this report refers to commercial tobacco products and not to sacred and traditional use of tobacco by certain American Indian communities. Initiation of tobacco product use during adolescence is associated with increased nicotine dependence and sustained tobacco product use into adulthood (*2*). Comprehensive tobacco control interventions have made substantial gains in decreasing tobacco product use among youths (*1*–*3*). Among U.S. high school students, current use of cigarettes declined from 36.4% in 1997 to 6.0% in 2019 (*3*). Although cigarette use among youths has declined, youths have engaged in the use of other tobacco products such as cigars, hookah, smokeless tobacco, and electronic vapor products (EVPs).

EVPs are known by many names including e-cigarettes, vapes, hookah pens, and mods (*2*). In 2018, the Surgeon General declared that e-cigarette use among youths had become an epidemic (*4*). EVPs use a heating element to aerosolize a liquid solution that users inhale. Vaping liquids come in a variety of flavors and typically contain nicotine, a highly addictive chemical that can affect brain development (*4*,*5*). Nicotine also might increase the likelihood of youths using combustible tobacco products and increase the risk for addiction to other substances (*4*,*5*). Moreover, EVPs can be used to deliver additional psychoactive substances such as tetrahydrocannabinol (THC) (*6*). In 2019, vitamin E acetate, an additive sometimes found in THC-containing EVPs, was linked to e-cigarette- or vaping product use-associated lung injuries (*6*).

Multiple factors influence the use of EVPs and other tobacco products among youths, such as targeted marketing to youths by the tobacco industry, the appealing flavors in EVPs, misperceptions that vaping relieves stress, peer and family influences, and low perceptions of harm (*1*–*5*,*7*). Other risk factors that prime youths for experimentation with tobacco products and other substances include social isolation, grief, trauma, and stress; these risk factors were commonly seen during the COVID-19 pandemic (*8*). The 2021 National Youth Tobacco Survey found that, among youths who currently use e-cigarettes, the most common reasons for use were feelings of anxiety, stress, or depression and the “high or buzz” associated with nicotine use (*7*).

Continued surveillance of tobacco product use among youths is crucial for guiding and evaluating tobacco prevention and control strategies at the local, state, tribal, and national levels. This report presents the latest data from the 2021 national Youth Risk Behavior Survey (YRBS) to assess ever, current, and daily use of EVPs among U.S. high school students and usual source of obtaining EVPs. This report also presents data from previous YRBSs (2015, 2017, and 2019) to examine trends in EVP use over time. These finding can be used to inform youth-focused tobacco prevention and control strategies at the local, state, tribal, and national levels.

## Methods

### Data Source

This report includes data from the 2015 (N = 15,624), 2017 (N = 14,765), 2019 (N = 13,677), and 2021 (N = 17,232) YRBSs, a cross-sectional, school-based survey conducted biennially since 1991. Each survey year, CDC collects data from a nationally representative sample of public and private school students in grades 9–12 in the 50 U.S. states and the District of Columbia. Additional information about YRBS sampling, data collection, response rates, and processing is available in the overview report of this supplement (*9*). The prevalence estimates for all tobacco product use questions for the entire study sample and stratified by sex, race and ethnicity, grade, and sexual orientation are available at https://nccd.cdc.gov/youthonline/App/Default.aspx. The full YRBS questionnaire, data sets, and documentation are available at https://www.cdc.gov/healthyyouth/data/yrbs/index.htm. This activity was reviewed by CDC and was conducted consistent with applicable federal law and CDC policy.*

### Measures

Student demographic characteristics analyzed included sex (female or male), sexual identity (heterosexual, gay or lesbian, bisexual, and other or questioning), and race and ethnicity. For sexual identity, the “other or questioning” category included students who selected, “I describe my sexual identity some other way” or “I am not sure about my sexual identity (questioning).” Students were classified into seven racial and ethnic categories, including American Indian or Alaska Native (AI/AN), Asian, Black or African American (Black), Native Hawaiian or other Pacific Islander (NH/OPI), White, Hispanic or Latino (Hispanic), and persons of multiple races (multiracial). (Persons of Hispanic origin might be of any race but are categorized as Hispanic; all racial groups are non-Hispanic.)

On the basis of how the questions were asked in the survey, electronic vapor products in this report refer to products “such as JUUL, SMOK, Suorin, Vuse, and blu” and include “e-cigarettes, vapes, vape pens, e-cigars, e-hookahs, hookah pens, and mods.” This study included four questions about EVP use, including ever use of EVPs, current EVP use (≥1 day during the 30 days before the survey), daily use of EVPs (during the 30 days before the survey), and usual source of EVPs (among youths who currently use EVPs) (Table 1).

**TABLE 1:** Question wording and analytic coding for included electronic vapor product variables* — Youth Risk Behavior Survey, United States, 2021

Variable	Question	Response options	Analytic coding	Years data available
Ever used an electronic vapor product	Have you ever used an electronic vapor product?	Yes, no	Yes versus no	2015–2021
Currently use electronic vapor products	During the past 30 days, on how many days did you use an electronic vapor product?	0 days, 1–2 days, 3–5 days, 6–9 days, 10–19 days, 20–29 days, or all 30 days	≥1 day versus 0 days	2015–2021
Daily use of electronic vapor products	During the past 30 days, on how many days did you use an electronic vapor product?	0 days, 1–2 days, 3–5 days, 6–9 days, 10–19 days, 20–29 days, or all 30 days	All 30 days versus 0–29 days	2015–2021
Usually got or bought their own electronic vapor products from a friend, family member, or someone else	During the past 30 days, how did you usually get your electronic vapor products? (Select only one response.)	I did not use any electronic vapor products during the past 30 days; I got or bought them from a friend, family member, or someone else; I bought them myself in a vape shop or tobacco shop; I bought them myself in a convenience store, supermarket, discount store, or gas station; I bought them myself at a mall or shopping center kiosk or stand; I bought them myself on the Internet, such as from a product website, vape store website, or other website like eBay, Amazon, Facebook Marketplace, or Craigslist; I took them from a store or another person; I got them in some other way	Got or bought them from a friend, family member, or someone else versus all other sources, among those who were current electronic vapor product users	2021

* The introduction to the electronic vapor products section of the YRBS questionnaire stated: “The next 3 questions ask about electronic vapor products, such as JUUL, SMOK, Suorin, Vuse, and blu. Electronic vapor products include e-cigarettes, vapes, vape pens, e-cigars, e-hookahs, hookah pens, and mods.”

### Analysis

Analyses were completed using SUDAAN (version 11.0.3; RTI International) to account for the complex survey design and weighting. Prevalence estimates and 95% CIs for questions assessing ever, current, and daily EVP use were calculated overall and for each sex, racial and ethnic, and sexual identity group. Statistically significant pairwise differences by demographic characteristics were determined by *t*-tests with Taylor series linearization. In addition, prevalence of behavior reported in 2021 was compared with the prevalence in 2019 by using *t*-tests with Taylor series linearization. Differences between prevalence estimates were considered statistically significant if the *t*-test p value was <0.05. Only statistically significant findings are described.

To identify temporal trends in EVP use, logistic regression analyses were used to model linear time effects while controlling for sex, grade (9, 10, 11, and 12), and race and ethnicity (*10*). EVP use was first introduced into the YRBS questionnaire in 2015; therefore, trends during 2015–2021 were examined for ever use of EVPs, current use of EVPs, and daily use of EVPs by sex and race and ethnicity. Students were presented with more response options for the question asking about sexual identity in 2021, which precludes the ability to examine trends in EVP use across sexual identity groups. Additional information about the methods used to conduct YRBS trend analyses are provided in the overview report of this supplement (*9*).

## Results

### Ever Used an Electronic Vapor Product

Overall, 36.2% of high school students ever used EVPs in 2021 (Table 2). The prevalence of ever use of an EVP varied by sex, race and ethnicity, and sexual identity. For example, the prevalence of EVP use was higher among female students (40.9%) than male students (32.1%); higher among Hispanic (40.4%), multiracial (36.8%), White (36.7%), NH/OPI (36.1%), Black (33.6%), and AI/AN students (33.5%) than Asian students (19.5%); and higher among bisexual students (48.9%) than heterosexual (34.7%), gay or lesbian (34.4%), and other or questioning students (33.5%).

**TABLE 2:** Prevalence of electronic vapor product* use among high school students, by sex, race and ethnicity, and sexual identity — Youth Risk Behavior Survey, United States, 2021

	Ever used an electronic vapor product^†^	Currently used electronic vapor products^§^	Daily use of electronic vapor products^¶^
	% (95% CI)**	% (95% CI)	% (95% CI)
**Total**	**36.2 (33.7**–**38.8)**	**18.0 (16.3**–**19.8)**	**5.0 (4.4**–**5.7)**
**Sex**			
Female	40.9 (37.6–44.2)	21.4 (19.2–23.8)	5.6 (4.6–6.8)
Male	32.1 (29.7–34.5)	14.9 (13.3–16.7)	4.5 (3.9–5.2)
**Race and ethnicity** ^††^			
American Indian or Alaska Native	33.5 (23.8–44.8)	23.2 (16.5–31.7)	4.4 (1.7–10.7)
Asian	19.5 (14.1–26.5)	5.5 (4.2–7.2)	1.2 (0.5–2.8)
Black	33.6 (30.4–37.0)	14.0 (12.3–16.0)	3.1 (2.0–4.7)
Native Hawaiian or other Pacific Islander	36.1 (29.2–43.7)	24.7 (17.2–34.3)	8.0 (3.6–16.8)
White	36.7 (34.2–39.3)	20.3 (18.4–22.2)	6.5 (5.6–7.6)
Hispanic/Latino	40.4 (36.7–44.2)	17.8 (15.3–20.5)	3.4 (2.9–4.1)
Multiracial	36.8 (30.9–43.2)	17.1 (13.4–21.5)	5.3 (4.0–6.8)
**Sexual identity**			
Heterosexual	34.7 (32.4–37.1)	16.4 (15.1–17.8)	4.4 (3.8–5.1)
Gay or lesbian	34.4 (25.5–44.6)	15.8 (11.1–22.0)	5.0 (2.9–8.6)
Bisexual	48.9 (44.2–53.6)	29.0 (25.4–32.8)	7.5 (5.7–9.9)
Other or questioning^§§^	33.5 (29.2–38.0)	15.7 (12.9–18.9)	4.6 (3.3–6.3)

*Refer to Table 1 for variable definitions.

^†^ On the basis of *t*-test analysis using Taylor series linearization, p<0.05. Responses from female students were significantly different than male students. Responses from Asian students were significantly different than American Indian or Alaska Native (AI/AN), Black, Hispanic, Native Hawaiian or other Pacific Islander (NH/OPI), White, and multiracial students. Responses from Black students were significantly different than Hispanic students. Responses from Bisexual students were significantly different than heterosexual, gay or lesbian, and other or questioning students.

^§^ On the basis of *t*-test analysis using Taylor series linearization, p<0.05. Responses from female students were significantly different than male students. Responses from Asian students were significantly different than AI/AN, Black, Hispanic, NH/OPI, White, and Multiracial students. Responses from Black students were significantly different than AI/AN, Hispanic, NH/OPI, and White students. Responses from bisexual students were significantly different than heterosexual, gay or lesbian, and other or questioning students.

^¶^ On the basis of *t*-test analysis using Taylor series linearization, p<0.05. Responses from Asian students were significantly different than Black, Hispanic, NH/OPI, White, and multiracial students. Responses from Black students were significantly different than White and multiracial students. Responses from Hispanic students were significantly different than White and multiracial students. Responses from White students were significantly different than multiracial students. Responses from bisexual students were significantly different than heterosexual and other or questioning students.

** N=17,232 respondents. Because the state and local questionnaires differ by jurisdiction, students in these schools were not asked all national YRBS questions. Therefore, the total number (N) of students answering each question varied. Percentages in each category are calculated on the known data.

^††^ Persons of Hispanic or Latino (Hispanic) origin might be of any race but are categorized as Hispanic; all racial groups are non-Hispanic.

^§§^ Includes students who responded, “I describe my sexual identity some other way” or “I am not sure about my sexual identity (questioning).”

During 2015–2021, a linear decrease occurred in ever use of an EVP (from 44.9% to 36.2%), overall and among male (from 46.1% to 32.1%), AI/AN (from 61.3% to 33.5%), Black (from 42.4% to 33.6%), Hispanic (from 51.9% to 40.4%), NH/OPI (from 61.4% to 36.1%), and multiracial students (from 48.1% to 36.8%) (Table 3). In addition, from 2019 to 2021, decreases were observed in ever use of an EVP, overall (from 50.1% to 36.2%), among female (from 50.7% to 40.9%), male (from 49.6% to 32.1%), AI/AN (from 57.9% to 33.5%), Black (from 40.0% to 33.6%), Hispanic (from 49.5% to 40.4%), NH/OPI (from 58.7% to 36.1%), White (from 54.7% to 36.7%), and multiracial students (from 55.3% to 36.8%).

**TABLE 3:** Trends in electronic vapor product use,* by sex and race and ethnicity — Youth Risk Behavior Survey, United States, 2015–2021

Behavior	2015^†^	2017^†^	2019^†^	2021^†^	Linear change 2015–2021^§^	Change during 2019–2021^¶^
**Ever used an electronic vapor product**						
**Total**	**44.9**	**42.2**	**50.1**	**36.2**	**Decreased**	**Decreased**
**Sex**						
Female	43.6	39.7	50.7	40.9	No linear change	Decreased
Male	46.1	44.9	49.6	32.1	Decreased	Decreased
Race and ethnicity**						
American Indian or Alaska Native	61.3	54.9	57.9	33.5	Decreased	Decreased
Asian	29.5	20.8	24.9	19.5	No linear change	No change
Black	42.4	36.2	40.0	33.6	Decreased	Decreased
Native Hawaiian or other Pacific Islander	61.4	49.1	58.7	36.1	Decreased	Decreased
White	43.2	41.8	54.7	36.7	No linear change	Decreased
Hispanic/Latino	51.9	48.7	49.5	40.4	Decreased	Decreased
Multiracial	48.1	46.8	55.3	36.8	Decreased	Decreased
**Currently use electronic vapor products**						
**Total**	**24.1**	**13.2**	**32.7**	**18.0**	**No linear change**	**Decreased**
**Sex**						
Female	22.6	10.5	33.5	21.4	Increased	Decreased
Male	25.6	15.9	32.0	14.9	Decreased	Decreased
**Race and ethnicity**						
American Indian or Alaska Native	30.2	27.8	47.3	23.2	No linear change	Decreased
Asian	14.5	3.7	13.0	5.5	Decreased	Decreased
Black	18.0	8.5	19.7	14.0	No linear change	Decreased
Native Hawaiian or other Pacific Islander	28.5	9.7	38.8	24.7	No linear change	Decreased
White	25.2	15.6	38.3	20.3	No linear change	Decreased
Hispanic/Latino	26.3	11.4	31.2	17.8	No linear change	Decreased
Multiracial	25.8	12.9	33.5	17.1	No linear change	Decreased
**Daily use of electronic vapor products**						
**Total**	**2.0**	**2.4**	**7.2**	**5.0**	**Increased**	**Decreased**
**Sex**						
Female	1.1	1.1	6.4	5.6	Increased	No change
Male	2.8	3.8	7.9	4.5	Increased	Decreased
**Race and ethnicity**						
American Indian or Alaska Native	3.2	7.7	11.5	4.4	No linear change	No change
Asian	1.2	0.6	3.0	1.2	No linear change	No change
Black	1.1	1.0	3.4	3.1	Increased	No change
Native Hawaiian or other Pacific Islander	5.4	3.1	5.4	8.0	No linear change	No change
White	1.9	3.1	9.3	6.5	Increased	Decreased
Hispanic/Latino	2.6	1.7	5.2	3.4	Increased	Decreased
Multiracial	2.8	2.6	8.2	5.3	Increased	No change

*Refer to Table 1 for variable definitions.

^†^ 2015: N = 15,624 respondents; 2017: N = 14,765 respondents; 2019: N = 13,677 respondents; 2021: N = 17,232 respondents. Because the state and local questionnaires differ by jurisdiction, students in these schools were not asked all national YRBS questions. Therefore, the total number (N) of students answering each question varied. Percentages in each category are calculated on the known data.

^§^ Logistic regression models were used to assess linear trends for 2015–2021, controlling for sex, race and ethnicity, and grade, p<0.05.

^¶^ On the basis of *t*-test analysis using Taylor series linearization, p<0.05.

** Persons of Hispanic or Latino (Hispanic) origin might be of any race but are categorized as Hispanic; all racial groups are non-Hispanic.

### Current Electronic Vapor Product Use

Overall, 18.0% of students currently used an EVP in 2021. The prevalence of current EVP use varied by sex, race and ethnicity, and sexual identity. For example, the prevalence of EVP use was higher among female students (21.4%) than male students (14.9%); higher among NH/OPI (24.7%), AI/AN (23.2%), White (20.3%), Hispanic (17.8%), multiracial (17.1%), and Black students (14.0%) than Asian students (5.5%); and higher among bisexual students (29.0%) than heterosexual (16.4%), gay or lesbian (15.8%), and other or questioning students (15.7%).

During 2015–2021, there was no linear change in current use of an EVP overall; however, there was a linear increase among female students (22.6% in 2015; 10.5% in 2017; 33.5% in 2019; 21.4% in 2021) and a linear decrease among male students (from 25.6% to 14.9%). There also was a linear decrease among Asian students (from 14.5% to 5.5%), but not among any other racial or ethnic group. From 2019 to 2021, decreases were observed in current use of an EVP overall (from 32.7% to 18.0%), among female (from 33.5% to 21.4%) and male (from 32.0% to 14.9%) students, and among AI/AN (from 47.3% to 23.2%), Asian (from 13.0% to 5.5%), Black (from 19.7% to 14.0%), Hispanic (from 31.2% to 17.8%), NH/OPI (from 38.8% to 24.7%), White (from 38.3% to 20.3%), and multiracial students (from 33.5% to 17.1%).

### Daily Use of Electronic Vapor Products

Overall, 5.0% of students reported daily use of an EVP in 2021. The prevalence of daily use of an EVP varied by race and ethnicity and sexual identity. For example, the prevalence of daily use of an EVP was higher among NH/OPI (8.0%), White (6.5%), multiracial (5.3%), Hispanic (3.4%), and Black students (3.1%) than among Asian students (1.2%). Prevalence also was higher among bisexual students (7.5%) than among other or questioning (4.6%) and heterosexual students (4.4%).

During 2015–2021, a linear increase occurred in daily use of an EVP overall (from 2.0% to 5.0%), among female (from 1.1% to 5.6%) and male students (from 2.8% to 4.5%), and among Black (from 1.1% to 3.1%), Hispanic (from 2.6% to 3.4%), White (from 1.9% to 6.5%), and multiracial students (from 2.8% to 5.3%). From 2019 to 2021, decreases were observed in daily use of an EVP use overall (from 7.2% to 5.0%), among male students (from 7.9% to 4.5%), and among Hispanic (from 5.2% to 3.4%) and White students (from 9.3% to 6.5%).

### Usual Source of Electronic Vapor Products

Among the 18.0% of students who currently used EVPs, 54.1% indicated they usually “got or bought them from a friend, family member, or someone else.” Other responses to the question about where students usually obtained EVP included, “bought them in a vape shop or tobacco shop” (12.4%), “bought them in a convenience store, supermarket, discount store, or gas station” (6.8%), “bought them at a mall or shopping center kiosk or stand” (0.5%), “bought them on the Internet, such as from a product website, vape store website, or other website like eBay, Amazon, Facebook Marketplace, or Craigslist” (1.7%), “took them from a store or another person” (2.8%), or “got them in some other way” (21.7%).

## Discussion

In 2021, more than one in three (36.2%) students had ever used EVPs and almost one in five (18.0%) students currently used EVPs. Overall, ever EVP use decreased and daily EVP use increased during 2015–2021; however, ever, current, and daily use of EVPs decreased from 2019 to 2021, a finding that is consistent with findings from other national surveillance systems, such as Monitoring the Future (*11*).

The 2021 YRBS documented variation in the patterns of EVP use between demographic groups. Prevalence of ever use and current use of EVPs were higher among female students than male students. During 2019–2022, adolescent females reported higher rates of eating disorders, emotional distress, anxiety, and depression related to the ongoing COVID-19 pandemic, all of which might explain increases in EVP use and other substances (*12*). Youths who engage in vaping behaviors as a method of dealing with stressors can potentially create a cycle of nicotine dependence because symptoms of nicotine withdrawal include symptoms of anxiety and depression (*1*).

This report disaggregated bisexual students from lesbian and gay students, revealing differences in EVP use among these two groups. Bisexual students had a higher prevalence of ever and current EVP use than lesbian or gay, other or questioning, or heterosexual students. This finding contributes to the evidence base that tobacco product usage among sexual minority (e.g., lesbian or gay, bisexual, and other or questioning [LGBQ+]) youths differs by sexual orientation (*13*).

EVP use was lower among Asian students in comparison with other racial and ethnic student groups. In general, prevalence of ever use, current use, and daily use of EVPs among Asian students was lower than among Black, Hispanic, NH/OPI, White, and multiracial students. This finding might be explained by other research that found social and cultural influences were protective factors against tobacco product and other substance use behaviors among Asian youths (*14*). Identifying risk and protective factors among youths is essential for developing tobacco prevention and cessation programs that address the various needs of youths.

It is concerning that daily EVP use increased among Black, Hispanic, multiracial and White students during 2015–2021. Observed patterns of increased daily use might be, in part, related to the increase in nicotine concentrations in U.S. e-cigarettes (*15*). A recent study found that, during 2013–2018, the average nicotine concentration in e-cigarettes sold increased by more than 80% for all flavor categories and rechargeable e-cigarettes (*15*). Exposure to nicotine during adolescence can affect learning, memory, and attention and increases risk for future nicotine dependence (*2*,*4*). Evidence-based cessation programs that are tailored and culturally specific to youths are needed to help youths who are nicotine dependent abstain from tobacco product usage.

Overall, ever use, current use, and daily use of EVPs among high school students decreased from 2019 to 2021. Certain factors might have contributed to this decline, including the implementation of policies restricting the sale of flavored tobacco products, and the COVID-19 pandemic, which provided youths fewer opportunities to purchase EVPs or interact with peers who use tobacco products and other substances (*4*,*11*,*12*). Regulatory efforts are ongoing at the national, state, and local levels to restrict youths access to EVPs and, thus, decrease the use of EVPs among youths. Findings from the 2021 YRBS found that among those who currently use EVPs, more than half (54.1%) got their EVPs from a friend, family member, or someone else, indicating students are finding other means to purchase or gain access to EVPs.

## Limitations

General limitations for YRBS are available in the overview report of this supplement (*9*). The findings in this report are subject to at least three additional limitations. First, the YRBS question addressing how students usually obtained EVPs limited respondents to only one response. Students might have obtained these products through multiple sources; therefore, the extent to which students use various sources are likely underrepresented. Second, EVP use as defined in this survey was not limited to vaping products that deliver nicotine. Therefore, results might overestimate nicotine-containing EVP use among youths. Finally, it was not possible to assess EVP use among subpopulations within the racial and ethnic categories included in this report (e.g., disaggregating students by racial and ethnic subgroups: Indian, Vietnamese, and Korean). Thus, the categories for race and ethnicity in this survey might not reflect the diversity of participants’ identities, and potentially masks nuanced differences in EVP use within racial and ethnic populations.

## Future Directions

The declines in ever, current, and daily EVP use among high school students during 2019–2021 is encouraging; however, prevalence of EVP use among students remains high. To reduce prevalence of EVP use among youths, public health professionals should consider using community-based participatory research to develop tobacco prevention and cessation programs that are tailored to youths (https://pubmed.ncbi.nlm.nih.gov/20147663). For example, an intervention was piloted among rural high school students in Kentucky where students were informed about the risks for e-cigarette use by their peers and provided cessation resources and information on how to help their friends abstain from cigarette use (https://www.ncbi.nlm.nih.gov/pmc/articles/PMC7789399). Results of that study yielded positive outcomes of the peer-led intervention related to increased awareness of risks associated with e-cigarette use and the desire of students to address the e-cigarette epidemic in their communities.

Future research could explore factors that influence EVP use among youths (e.g., neighborhood poverty or socioeconomic status), access to health care, access to healthy food, and opportunities for physical activity. Such factors can be conceptualized as social determinants of health that might influence EVP use among students. Addressing social determinants of health when developing youth-centric tobacco control programs could improve evidence-based interventions to reduce tobacco product use initiation among students and provide tailored cessation services to youths who use tobacco products.

Future research could explore EVP use among youths with identities not examined in this report. There is a dearth of research describing EVP use among nonbinary, gender fluid, gender expansive youths (https://www.edi.nih.gov/people/sep/lgbti/safezone/terminology); those whose sexual orientation is not captured by the categories of “lesbian,” “gay” or “bisexual”; and youths with intersectional identities including those who are LGBQ+ and of certain racial and ethnic minority groups. Future research that uses an intersectional approach to understanding EVP use among LGBQ+ youths and youths of various racial and ethnic identities can inform evidence-based tobacco control interventions and promising practices for persons most at risk for EVP use.

Finally, approximately half of high school youths who currently use EVPs were getting or buying these products from a friend, family member, or someone else. These findings provide an opportunity to use practices that focus on the social influences of tobacco product usage among youths. Programs like the Truth Initiative’s *This is Quitting* (https://truthinitiative.org/thisisquitting) are tailored to youths to address the social and behavioral factors that lead to EVP and other tobacco product use. Evidence-based interventions at the individual- and community-level can provide tools to youths that address peer pressure, encourage self-efficacy and goal-setting, and increase the knowledge base of EVP use and associated harms to reduce or eliminate tobacco product use among youths (https://catch.org/wp-content/uploads/2021/05/SAMHSA-CATCH-My-Breath-Reducing-Vaping-Among-Youth-and-Young-Adults.pdf).

## Conclusion

EVP use among U.S. high school students remains a public health concern. During 2015–2021, no linear decrease was observed in current EVP use among high school students overall; a linear increase was observed among female students who reported current EVP use. In addition, daily EVP use increased overall and among female, male, Black, Hispanic, multiracial, and White students during 2015–2021. Eliminating EVP use among youths requires evidence-based strategies and practices that are culturally relevant and tailored to the communities most at risk for sustained EVP use. Continued surveillance of EVP use among youths is necessary to guide and evaluate public health strategies at the local, state, tribal and national levels.

## Footnotes

* See e.g., 45 C.F.R. part 46.102(l)(2), 21 C.F.R. part 56; 42 U.S.C. §241(d); 5 U.S.C. §552a; 44 U.S.C. §3501 et seq.
